# It takes a village: Community-based organizations and the availability and utilization of HIV/AIDS-related services in Nigeria

**DOI:** 10.1080/09540121.2012.740158

**Published:** 2013-06-09

**Authors:** Jakub Kakietek, Tesfayi Geberselassie, Brigitte Manteuffel, Kayode Ogungbemi, Anya Krivelyova, Sarah Bausch, Rosalía Rodriguez-García, Rene Bonnel, N'Della N'Jie, Joseph Fruh, Sani Gar

**Affiliations:** a ICF International, Atlanta, GA, USA; b National Agency for the Control of AIDS, Abuja, Nigeria; c The World Bank, Washington, DC, USA; d National Population Commission, Abuja, Nigeria

**Keywords:** HIV, community, community-based organization (CBO), prevention, treatment, Nigeria

## Abstract

Community-based organizations (CBOs) have emerged as a vital part of the response to HIV/AIDs in Nigeria. The evaluation, on which this article is based, conducted in 28 communities in 6 states and the Federal capital Territory in Nigeria, assessed the effects of the CBO engagement on a set of outcomes related to HIV/AIDS knowledge, attitudes, beliefs, and practices, stigma, service availably and utilization and social capital. It consisted of three components: a household survey conducted in all 28 communities, qualitative data collected from CBO staff and key informants (KIs), and a funding allocation study (qualitative interviews and the funding allocation study were conducted in a subset of 14 communities). This article focuses on the association between CBO engagement and reported availability and utilization of HIV/AIDS-related services. It shows that CBO engagement has a potential to add value to the national response to HIV/AIDS by increasing the awareness, availability, and utilization of HIV/AIDS-related services, especially in the area of prevention, care and support. The CBOs in the evaluation communities focused on prevention activities as well as on providing support for people living with HIV/AIDS (PLWHA) and prevention and care and support were the highest expenditure categories reported by CBOs. Respondents in communities with a stronger CBO engagement were more likely to: (1) be aware of any HIV/AIDs-related services, (2) report that prevention and care services were available in their communities, and (3) have used any HIV/AIDS related services, prevention-related and care-related services than respondents in communities where CBO engagement was weaker. The association between service awareness and service use and CBO engagement was stronger in rural than in urban areas.

## Introduction

Over the last decade, national governments and international donors have recognized the potential of community-based organizations (CBOs) in the fight against HIV/AIDS (Global Fund to Fight AIDS, Tuberculosis, & Malaria, 2010; Schwartländer et al, 2011; UNAIDS, 2011;). This recognition, and corresponding increases in the funds allocated to CBOs are based on the assumption that responding to the epidemic is best achieved by the active involvement of local communities. CBOs are thought to be more flexible and adaptable than governmental agencies due to their capability to use grassroots approaches and mobilize community members, and reach rural, marginalized, or politically disenfranchised populations (Clark, 1995; Lewis & Wallace, 2000; Streeten, 1997; Warkentin, 2001). They are also often considered more efficient than governmental or intergovernmental multilateral agencies (e.g., the UN family organizations) because they operate with lower overhead costs and less bureaucratic red tape (Clark, 1995).

To test this assumption and provide empirical evidence, the World Bank, the Department for International Development (DFID), and the Nigerian National Agency for the Control of AIDS (NACA) commissioned a scientifically rigorous evaluation of the community response in Nigeria. The evaluation assessed the relationship between CBO engagement at the community level and a wide range of outcomes including HIV/AIDS knowledge, stigma and attitudes vis-à-vis people living with HIV/AIDS (PLWHAs), availability and utilization of HIV/AIDS-related services, gender norms, and social capital. This article focuses on the association between CBO engagement and service availability and use. It aims at answering the following question: Do community members in communities with a stronger CBO engagement in the response to HIV/AIDS have greater access HIV/AIDS-related services and are more likely to use those services compared to community members in communities with a weaker CBO engagement?

### Context

The first HIV case was identified in Nigeria in 1986 (National HIVAIDS Policy, 2005–2009) and currently, the country is home to the second largest population of PLWHA, behind South Africa (Monjok, Smesny, & Essien, 2009). The overall adult seroprevalence rate is 3.7%, and varies greatly by geo-political region, ranging from 2.0% in the South West to 7.0% in the South South, and by age group, ranging from 2.9% in 40–44 year olds to 5.6% in 25–29 year olds (UNAIDS, 2010). Sexual intercourse remains the most common mode of HIV transmission (80%), followed by mother-to-child transmission (10%) and infected blood and blood products (10%). Nearly 40% of new infections are attributable to high-risk populations, including injecting drug users, female sex workers, and men who have sex with men (NACA, 2007).

CBOs have emerged as a vital part of the response to HIV/AIDs. They have engaged in in ancillary care (e.g., social support, referrals) and treatment (e.g., antiretrovirals, treatment for opportunistic infections; UNAIDS, 2008). They have also become a key partner to the government in developing, implementing, and monitoring the national response to AIDS (UNAIDS, 2005). Through the Civil Society Consultative Group on HIV/AIDS in Nigeria (CiSCGHAN), established in 2002, CBOs provide input in policy formulation and development. CiSCGHAN is involved in the consultation process of the World Bank Multi-Country HIV/AIDS program to ensure the World Bank HIV/AIDS Fund (HAF) reflects the needs of the civil society. Some of the international funding (e.g., HAF) are channeled directly to CBOs that provide prevention, care, and support services in local communities.

## Methods

The evaluation was conducted in 28 communities in 6 states: Adamawa, Akwa Ibom, Benue, Enugu, Kaduna, and Lagos (four communities per state), representing Nigeria's six geopolitical zones. It consisted of three components: a household survey, in- depth interviews with CBO staff and community key informants (KIs), and a funding allocation study (in-depth interviews and the funding allocation study were conducted in the subset of 14 communities). The household survey was administered to a sample of 5376 households (192 per community) which were randomly selected within community clusters. In each household, the head of the household was asked questions pertaining to the household and a randomly selected adult member of the household (who sometimes was also the household head) was asked questions regarding knowledge, attitudes, and beliefs regarding HIV/AIDS, past sexual behavior, service availability and use. Table 1 presents the basic demographic characteristics of the survey sample:

**Table 1.** Demographic characteristics of the Household Survey sample.

		*N*	%
Residence	Urban	3033	56.9
	Rural	2298	43.1
Gender	Male	2670	50.1
	Female	2657	49.9
Marital Status	Married or living together	3704	69.5
	Divorced/separated	88	1.7
	Widowed	174	3.3
	Never married	1361	25.6
Education	No secondary education	1869	34.9
	High school/vocational education	2164	40.7
	Some college or university education	1301	24.4
Engaged in any paid work	Yes	3931	77.2
	No	1163	22.8
		Mean	SD
Age		34.4	10.7

An average respondent in the sample was 34.4 years old and the sample consisted of equal proportions of females and males (49.9% and 50.1%, respectively). About 56.9% of the respondents resided in urban areas, 69.5% were married or had a live-in partner, 25.6% had never been married, 3.3% were widowed, and 1.7% were divorced/separated. About 34.9% of the respondent did not have any secondary education, 40.7% of the respondents completed high school/had secondary vocational education, and 24.39% had some college or university education. 77.2% of the respondents engaged in paid work.

In-depth interviews conducted with staff from a total of 69 CBOs provided information regarding activities and programs in which they engaged. The funding allocation component provided information about the annualized funding received by the CBOs and their expenditures, as well as per-persons cost of services they provided. On average, staff members of 59% of all CBOs active in a given community were interviewed.

The interviews with community KIs were designed to obtain an assessment of CBO engagement from the point of view of the community members and information about the broader community context. A total of 66 KIs was interviewed (23 health sector workers, 31 community and religious leaders, and 12 principals or teachers).

The multi-method multi-component design enabled the research team to triangulate information from all three evaluation components, by mapping CBO activities and expenditures, and assessing the association between CBO engagement and individual- level outcomes reported in the household survey.

### Measures

Community was defined as a collection of household units made up of at least 5000 people (or 1000 households) living in the same geographical area. This definition reflects the operational definition used in the Nigerian Strategic Framework (NACA, 2007). A CBO was defined as a service organization with strong community involvement that provides services aimed at helping people infected with and affected by HIV/AIDS. The strength of CBO engagement in the community response to HIV/AIDS was defined as the extent to which local CBOs were involved in the response to the epidemic in their communities by providing services to community members. It was operationalized based on the number of CBOs present in the community.

The communities included in the evaluation were paired, so that, in each state, there was an urban and a rural pair.^1^ Within each pair, one of the selected communities had a higher number of CBOs than the other community. This approach allowed for identifying and including in the evaluation communities with a significant variation in the number of CBOs.

CBO engagement was measured by the number of CBOs per 100,000 inhabitants. The higher number of CBOs per 100,000 population was interpreted as stronger CBO engagement. This indicator reflected the concept of CBOs engagement as the extent to which CBOs had a potential to interact with and affect community members. It ranged from 0 to 7.17 and an average community had about 3 CBOs per 100,000 population (mean: 2.7; standard deviation: 2.12; a continuous variable was used in all analyses). Because population figures were not available at the community level, population figures for the Local Government Areas where the evaluation communities were located were used.

Measures of service availability and utilization were based on the survey data. Survey participants were asked whether any HIV/AIDS related-services, prevention services, treatment services, and care and support services were available in their community and whether they used services in any of those categories. Confirmatory responses were coded as 1 and non-confirmatory as 0.

### Analysis

The effects of community engagement on HIV/AIDS-related outcomes were assessed using multiple logistic regression analysis. Because the sample had a multi-level structure, with household nested within communities, and communities nested within states, hierarchical linear modeling was used. The following control variables were included to account for potential confounding: respondent's age, sex, education, marital status, employment status, exposure to media, household wealth index (individual-level covariates), and HIV prevalence and rural/urban character of the community (community-level covariates). In addition, the pair-wise indicator of CBO presence used to select the evaluation sample was included as a control variable. A random intercept (a community- specific random effect) accounted for the clustering of observations at the community level.

Initial review and descriptive analysis of the survey data suggested that the association between service use and awareness and the strength of CBO engagement may be different in urban and in rural areas. To confirm this hypothesis, the multivariate models used to analyze the full sample were re-estimated on two sub-samples, one containing only rural respondents and the other only urban respondents.

All models were fitted using Stata version 11 and its GLLAMM module for generalized linear latent and mixed models (StataCorp., 2009). Separate regression models were estimated for availability and use of each service category.

## Results

Analysis of the interviews with CBO staff showed that the CBOs focused on prevention and support activities: 77% of all interviewed CBOs engaged in some type of prevention efforts, 39% in support activities targeting PLWHA, and 42% in support activities targeting orphans and vulnerable children (OVC) (see Table 2). The emphasis on prevention and support activities was visible in all communities included in the evaluation and CBOs in communities with higher and lower engagement offered a similar range of services and activities.

**Table 2.** Average number and percentage of interviewed CBOs per community engaging in different types of HIV/AIDS activities.

Activity area	Total *N*	%
Prevention (e.g., education/information campaign, VCT, condom distribution)	53	77
Treatment (e.g., ART, opportunistic infections)	12	17
Care (e.g., home based care)	19	27
Support (e.g., support groups)	27	39
Impact Mitigation (e.g., income-generating activities)	17	25
Advocacy (e.g., community campaigns)	13	19
Support for OVC (e.g., scholarships, help buying school supplies)	29	42

Note: Data based on semi-structured interviews with CBO staff.

Consistently, the CBOs spent most of their funds on prevention services and socioeconomic impact mitigation (including support services for PLWHA and OVC) (25% and 23% of total expenditure, respectively).

Association between CBO engagement and the reported availability and utilization of HIV/AIDS-related service

**Figure 1.**
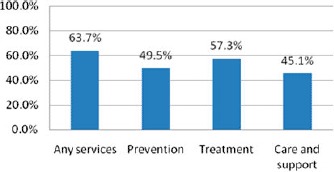
Reported service awareness and availability in the evaluation sample, by service category. Notes: “Prevention services” category included: information and awareness raising, life skills, behavior change communication, action to change harmful traditional practices, stigma reduction, activities to change cultural and gender norms to reduce stigma and discrimination; condom distribution, provisions of needles and bleach, HIV testing and counseling, HIV testing promotions, outreach to groups at risk; “Treatment services” category included: provision of ART, visits to health facilities, referral to health facilities, support for HIV and TB treatment adherence, treatment education and literacy, mother-to-child transmission prophylaxis; “Care and suppor”: category included: social, psychological, and spiritual support, counseling, childcare, day and respite care, home-based care, palliative care, nutrition support, support for OVC, support groups and self-help activities; “Any services” included any of the services mentioned earlier. Percentages are based on self-reports from the household survey.

Overall, 63.7% of the survey respondents said that HIV/AIDS related services were available in their community. About 57.3% of the respondents said that treatment services were available, 45.1% said that prevention services were available, 49.5% said that care services were available in their community (data based on self-reports) (see Figure 1).

Stronger CBO engagement (measured as the number of CBOs per 100,000 population) was associated with greater reported availability of prevention and care services (see Table 2). An increase of 1 in the number of CBOs per 100,000 was associated with a 48% increase in the odds of the respondent's reporting that prevention services were available in the community (aOR: 1.48; 95%CI:1.12–1.96) and 33% increase in the odds of the respondent's reporting that care services available in the community (aOR: 1.33; 95%CI: 1.01–1.76) (see Table 2).

Men were more likely than women to report the availability of any services, treatment services, care services and prevention services, and respondents with higher educational attainment, respondents from wealthier households and respondents who were employed were more likely to report that any services, prevention, treatment, and care and support services were available in their community than respondents who had less education, were unemployed or came from poorer households.

**Figure 2.**
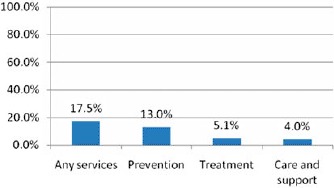
Reported service utilization in the evaluation sample, by service category. Note: “Prevention services” category included: information and awareness raising, life skills, behavior change communication, action to change harmful traditional practices, stigma reduction, activities to change cultural and gender norms to reduce stigma and discrimination; condom distribution, provisions of needles and bleach, HIV testing and counseling, HIV testing promotions, outreach to groups at risk; “Treatment services” category included: provision of ART, visits to health facilities, referral to health facilities, support for HIV and TB treatment adherence, treatment education and literacy, mother-to-child transmission prophylaxis; “Care and support” category included: social, psychological, and spiritual support, counseling, childcare, day and respite care, home-based care, palliative care, nutrition support, support for OVC, support groups and self-help activities; “Any services” included any of the services mentioned earlier. Percentages are based on self-reports from the household survey.

In contrast to reported service availability, utilization of HIV/AIDS related services was low. Only 17.5% of respondents reported using any services, 13% reported using prevention services, 5.1% reported using treatment services, and 4% reported using care services. Furthermore, in 7 out of the 28 communities included in the evaluation, none of the respondents reported using any HIV/AIDS-related services (Figure 2).

In the multivariate analyses, a greater number of CBOs per 100,000 was associated with greater use of any services, prevention services, and care services. An increase of one in the number of CBOs per 100,000 was associated with over a two-fold increase in the odds that a respondent reported using any services (aOR: 2.06; 95% CI: 1.21–3.50), a 2.5-fold increase in the odds of reporting using care services (aOR: 2.49; 95% CI: 1.16–5.33), and over a four-fold increase in the odds of reporting using prevention services (aOR: 4.39; 95% CI: 1.56–12.35). In addition to the number of CBOs, household wealth, respondents’ education, employment, and marital status were associated with service use (see Table 3).

**Table 3.** Results of multi-level regression analysis: services awareness and reported service availability.

	Are you aware of any HIV/AID-related in this community?		Are any services in the following categories available in your community?					
			Prevention		Treatment		Care and support	
	aOR	95% CI	aOR	95% CI	aOR	95% CI	aOR	95% CI
CBOs per 100,000	1.35	(0.99–1.83)	**1.48**	**(1.12–1.96)**	1.31	(0.98–1.75)	**1.33**	**(1.01–1.76)**
Gender	**0.71**	**(0.61–0.83)**	**0.8**	**(0.68–0.93)**	**0.67**	**(0.57–0.77)**	**0.66**	**(0.57–0.76)**
Age	**1.01**	**(1.00–1.02)**	**1.01**	**(1.00–1.02)**	**1.01**	**(1.00–1.02)**	**1.01**	**(1.00–1.02)**
Education								
Secondary	**1.49**	**(1.24–1.79)**	**1.63**	**(1.36–1.96)**	**1.35**	**(1.13–1.61)**	**1.29**	**(1.09–1.54)**
College/university	**1.75**	**(1.39–2.19)**	**1.84**	**(1.48–2.29)**	**1.63**	**(1.31–2.02)**	**1.61**	**(1.31–1.98)**
Employed	**1.57**	**(1.31–1.89)**	**1.41**	**(1.17–1.69)**	**1.53**	**(1.28–1.82)**	**1.43**	**(1.20–1.70)**
Marital status								
Married	1.13	(0.92–1.39)	0.98	(0.80–1.19)	1.17	(0.97–1.43)	1.04	(0.86–1.26)
Widowed/separated	0.84	(0.58–1.22)	0.55	(0.37–0.81)	0.83	(0.58–1.20)	0.62	(0.43–0.89)
Exposure to mass media	1.05	(0.86–1.28)	0.74	(0.61–0.91)	1.07	(0.88–1.29)	0.98	(0.81–1.18)
Household wealth index	**1.68**	**(1.47–1.92)**	**1.52**	**(1.34–1.74)**	**1.71**	**(1.50–1.95)**	**1.64**	**(1.44–1.87)**
HIV prevalence	1.03	(0.92–1.14)	0.99	(0.91–1.08)	1.04	(0.94–1.15)	1.02	(0.93–1.13)
Rural/urban	1.3	(0.39–4.37)	0.78	(0.28–2.16)	1.58	(0.50–4.98)	2.05	(0.70–6.05)
Pair-wise assignment	0.58	(0.17–1.93)	0.58	(0.18–1.82)	0.68	(0.22–2.13)	0.52	(0.18–1.53)
*N*	5170		5170		5170		5170	
Level 2 variance	2.05		2.07		1.85		1.71	
Level 2 variance (s.e.)	0.6		0.58		0.54		0.55	

Notes: All coefficients significant at the 95% level are in bold font. The estimated conditional models were specified as follows: DV_ij_ = γ_00_ + ß_11_Age_ij_ + ß_12_Gender_ij_ + ß_13_Marital status_ij_ + ß_14_ Empolyment status_ij_ + ß_15_ Exposure to media + ß_16_ Househod wealth index_j_ + γ_01_CBO density_j_ + γ_02_ Pair-wise assignment_j_ + γ_03_HIV prevalence_j_ + γ_06_ Rural/urban_j_ + u_0j_ + r_ij_ where DV_ij_ is the dependent variable from ith individual in the *j*th community; γ_00_ is the non-random intercept term; ß_ij_ is the coffecient for the ith individual-level variable from *j*th community; γ_0j_ is the coefficient for the *j*th community-level variable; u_0j_ is the community-level residual; and r_ij_ is the individual-level residual.

### Difference between rural and urban areas

The association between service awareness and service use and CBO engagement was stronger in rural than in urban areas. In rural areas, an increase of one in the number of CBOs per 100,000 was associated with 44% increase in the odds that the respondent reporting that any HIV/AIDS services were available in her or his community (aOR: 1.44; 95%CI: 1.35–1.54), compared to only 19% increase in the odds in urban areas (aOR: 1.19, 95%CI: 1.12–1.25; see Table 4). Similarly, in rural areas, an increase of one in the number of CBOs per 100,000 was associated with 48% increase in the odds of the respondent reporting that prevention services and were available in her or his community (aOR: 1.48; 95%CI: 1.40–1.57) and 31% increase in the odds of reporting that treatment services were available (aOR: 1.31; 95%CI: 1.24–1.38), compared to 26% (aOR: 1.26; 95%CI: 1.21–1.33) and 16% (aOR1.16%; 95%CI: 1.10–1.22) increase, respectively, in urban areas. The estimates of the association between the number of CBOs and awareness of care services, were almost identical in rural and urban areas (aOR 1.31; 95%CI: 1.24–1.40; aOR 1.21; 95%CI: 1.14–1.26) (Table 5).

**Table 4.** Results of multi-level regression analysis: reported service utilization.

	Have you use any of the HIV/AIDS related service categories in the past 12 months							
	Any HIV/AIDS services		Prevention		Treatment		Care and support	
	aOR	95% CI	aOR	95% CI	aOR	95% CI	aOR	95% CI
CBOs per 100,000	**2.06**	**(1.21–3.50)**	**4.39**	**(1.56–12.35)**	**1.43**	(0.83–2.47)	**2.49**	**(1.16–5.33)**
Gender	0.96	(0.78–1.17)	0.82	(0.65–1.03)	1.23	(0.92–1.65)	0.48	(0.34–0.67)
Age	0.98	(0.97–1.00)	1	(0.99–1.01)	**0.96**	**(0.95–0.98)**	0.99	(0.97–1.01)
Education								
Secondary	1.15	(0.88–1.49)	**1.5**	**(1.12–2.02)**	**0.63**	**(0.43–0.92)**	1.5	(0.91–2.47)
College/university	**1.79**	**(1.33–2.41)**	**2.66**	**(1.90–3.73)**	0.8	(0.53–1.22)	**2**	**(1.18–3.40)**
Employed	1.28	(0.98–1.68)	1.08	(0.79–1.48)	1.25	(0.86–1.83)	**1.67**	**(1.03–2.70)**
Marital status								
Married	**1.99**	**(1.53–2.60)**	**1.71**	**(1.27–2.29)**	**1.56**	**(1.08–2.28)**	**1.63**	**(1.07–2.49)**
Widowed/separated	1.64	(0.97–2.76)	0.99	(0.54–1.80)	**3.55**	**(1.81–6.95)**	0.57	(0.16–2.03)
Exposure to mass media	0.93	(0.69–1.24)	0.91	(0.66–1.26)	1.43	(0.91–2.24)	1.36	(0.78–2.38)
Household wealth index	**1.38**	**(1.16–1.65)**	**1.41**	**(1.14–1.75)**	1.14	(0.90–1.44)	1.24	(0.94–1.64)
HIV prevalence	0.87	(0.69–1.09)	0.8	(0.56–1.14)	0.93	(0.75–1.15)	0.85	(0.64–1.15)
Rural/urban	0.6	(0.07–4.89)	0.05	(0.00–1.85)	2.44	(0.26–22.65)	0.3	(0.02–5.36)
Community assignment	0.71	(0.07–6.89)	0.2	(0.01–6.00)	1.14	(0.13–10.26)		0.23-(0.01
Constant	Na		Na		Na		Na	
*N*	5170		5170		5170		5170	
Level 2 variance	6.88		15.4		5.14		7.17	
Level 2 variance(s.e.)	2.22		8.51		2.09		3.61	

Notes: All coefficients significant at the 95% level are in bold font. The estimated conditional models were specified as follows: DV_ij_ = γ_00_+ß_11_ Age_ij_ + ß_12_ Gender_ij_ + ß_13_ Marital status_ij_ + ß_14_ Empolyment status_ij_+ ß_15_ Exposure to media + ß_16_ Househod wealth index_j_ + γ_01_CBO density_j_ + γ_02_ Pair-wise assignment_j_ + γ_03_ HIV prevalence_j_ + γ_06_ Rural/urban_j_ + u_0j_ + r_ij_ where DV_ij_ is the dependent variable from ith individual in the *j*th community; γ_00_ is the non-random intercept term; ß_ij_ is the coffecient for the ith individual-level variable from *j*th community; γ_0j_ is the coefficient for the *j*th community-level variable; u_0j_ is the community-level residual; and r_ij_ is the individual-level residual.

**Table 5.** Results of multi-level regression analysis: services awareness and reported service availability in rural and urban communities.

	Are you aware of any services for people with HIV in this community?				Any prevention services available			
	Rural		Urban		Rural		Urban	
	aOR	95% CI	aOR	95% CI	aOR	95% CI	aOR	95% CI
CBOs per 100,000	**1.44**	**(1.35–1.54)**	**1.19**	**(1.12–1.25)**	**1.48**	**(1.40–1.57)**	**1.26**	**(1.21–1.33)**
Gender	**0.61**	**(0.49–0.76)**	**0.8**	**(0.66–0.96)**	**0.64**	**(0.51–0.80)**	**0.91**	**(0.77–1.08)**
Age	1.00	(0.99–1.01)	1.02	(1.00–1.03)	1.00	(1.00–1.02)	1.01	(1.00–1.02)
Education								
Secondary	1.06	(0.84–1.32)	**1.27**	**(1.01–1.61)**	0.96	(0.76–1.22)	**1.3**	**(1.04–1.62)**
College/university	1.44	(0.95–1.49)	**1.68**	**(1.31–2.17)**	**1.55**	**(1.14–2.10)**	**1.8**	**(1.42–2.27)**
Employed	**1.19**	**(1.18–2.08)**	0.95	(0.76–1.18)	1.14	(0.89–1.46)	0.88	(0.72–1.07)
Marital status								
Married	**1.57**	**(1.18–2.08)**	0.95	(0.75–1.20)	1.14	(0.86–1.51)	0.82	(0.66–1.02)
Widowed/divorced/separated	1.07	(0.62–1.86)	**0.6**	**(0.39–0.94)**	0.98	(0.56–1.74)	**0.37**	**(0.23–0.57)**
Household wealth index	**1.56**	**(1.42–1.71)**	**0.44**	**(0.35–0.56)**	**1.61**	**(1.45–1.78)**	**0.44**	**(0.37–0.54)**
HIV prevalence	**1.07**	**(1.04–1.09)**	0.99	(0.98–1.01)	**1.06**	**(1.04–1.09)**	**0.97**	**(0.96–0.98)**
Exposure to mass media	1.15	(0.89–1.49)	1.04	(0.82–1.32)	0.93	(0.70–1.22)	1.05	(0.84–1.31)
Community assignment	**0.30**	**(0.23–0.38)**	1.2	(0.98–1.39)	**0.22**	**(0.17–0.29)**	**1.39**	**(1.18–1.64)**
*N*	2226		2944		2226		2944	
	Any treatment services available?				Any care and support services available			
CBOs per 100,000	**1.31**	**(1.24–1.38)**	**1.16**	**(1.10–1.22)**	**1.18**	**(1.12–1.24)**	**1.21**	**(1.14–1.26)**
Gender	**0.63**	**(0.51–0.78)**	**0.67**	**(0.56–0.80)**	**0.56**	**(0.45–0.69)**	**0.67**	**(0.56–0.79)**
Age	1.00	(0.99–1.01)	1.01	(1.00–1.02)	1.00	(0.99–1.02)	1.00	(0.99–1.01)
Education								
Secondary	1.08	(0.86–1.34)	1.16	(0.93–1.45)	0.89	(0.71–1.13)	1.03	(0.82–1.27)
College/university	**1.54**	**(1.15–2.08)**	**1.64**	**(1.13–2.08)**	1.19	(0.87–1.62)	**1.52**	**(1.21–1.91)**
Employed	1.17	(0.93–1.47)	0.88	(0.71–1.08)	1.11	(0.87–1.42)	**0.79**	**(0.65–0.97)**
Marital status								
Married	**1.52**	**(1.16–2.00)**	1.03	(0.82–1.28)	**1.55**	**(1.16–2.07)**	**1.13**	**(0.91–1.40)**
Widowed/divorced/separated	0.82	(0.47–1.41)	0.73	(0.47–1.12)	0.61	(0.33–1.14)	0.71	(0.47–1.08)
Household wealth index	**1.48**	**(1.34–1.62)**	**0.59**	**(0.49–0.72)**	**1.54**	**(1.40–1.70)**	**0.63**	**(0.53–0.76)**
HIV prevalence	**1.04**	**(1.02–1.07)**	1.00	(0.99–1.02)	**1.07**	**(1.04–1.09)**	**0.98**	**(0.97–0.99)**
Exposure to mass media	1.15	(0.89–1.48)	1.02	(0.81–1.13)	1.02	(0.78–1.33)	1.08	(0.86–1.35)
Community assignment	0.61	(0.49–0.77)	1.07	(0.90–1.26)	0.28	(0.21–0.89)	1.01	(0.85–1.20)
N	2226		2944		2226		2944	

Notes: All coefficients significant at the 95% level are in bold font. The estimated conditional models were specified as follows: DV_ij_ = γ_00_+ß_11_ Age_ij_ + ß_12_ Gender_ij_ + ß_13_ Marital status_ij_ + ß_14_ Empolyment status_ij_+ ß_15_Exposure to media + ß_16_ Househod wealth index_j_ + γ_01_CBO density_j_ + γ_02_ Pair-wise assignmentj + γ_03_ HIV prevalence_j_ + u_0j_ + r_ij_ where DV_ij_ is the dependent variable from ith individual in the *j*th community; γ_00_ is the non-random intercept term; ß_ij_ is the coffecient for the ith individual-level variable from *j*th community; γ_0j_ is the coefficient for the *j*th community-level variable; u_0j_ is the community-level residual; and r_ij_ is the individual-level residual.

In rural communities, an increase of one in the number of CBOs per 100,000 was associated with a two-fold increase in the odds of respondent reported using any HIV/AIDS services (aOR: 2.08; 95%CI: 1.93–2.26) compared to only 22% increase in urban communities (aOR: 1.22; 95%CI: 1.16–1.28). Similarly, in rural communities, an increase of one in the number of CBOs per 100,000 was associated with a two-fold increase in the odds that a respondent reported using prevention services (aOR: 2.09; (%%CI: 1.91–2.28) compare to only 24% increase in the odds in urban communities. The use of treatment services was significantly associated with the number of CBOs in rural areas but not in urban areas (aOR: 1.64; 95%CI: 1.51–1.80 and aOR: 0.94; 95%CI: 0.94–1.08). Association between the number of CBOs and utilization of care services had in rural and urban areas was of similar magnitude (aOR: 1.65; 95%CI: 1.51–1.80, and aOR: 1.66; 95%CI: 1.49–1.91, respectively) (Table 6).

**Table 6.** Results of multi-level regression analysis: reported service utilization in rural and urban communities.

	Do you use any HIV-related services				Use prevention services			
	Rural		Urban		Rural		Urban	
	aOR	95% CI	aOR	95% CI	aOR	95% CI	aOR	95% CI
CBOs per 100,000	**2.08**	**(1.93–2.25)**	**1.22**	**(1.16–1.28)**	**2.09**	**(1.91–2.28)**	**1.24**	**(1.18–1.31)**
Gender	1.24	(0.91–1.70)	1.12	(0.91–1.39)	0.87	(0.62–1.23)	1.08	(0.85–1.37)
Age	0.99	(0.97–1.01)	1.01	(0.99–1.02)	1.01	(0.99–1.03)	1.01	(1.00–1.03)
Education								
Secondary	**1.69**	**(1.16–2.45)**	0.93	(0.70–1.22)	2.11	(1.37–3.24)	0.91	(0.66–1.25)
College/university	**3.77**	**(2.44–5.84)**	**1.48**	**(1.11–1.96)**	**4.62**	**(2.81–7.59)**	**1.59**	**(1.15–2.19)**
Employed	**2.32**	**(1.51–3.55)**	**1.32**	**(1.03–1.70)**	**1.91**	**(1.18–3.08)**	1.2	(0.91–1.58)
Marital status								
Married	1.28	(0.87–1.89)	**0.75**	**(0.58–0.98)**	1.34	(0.87–2.06)	**0.69**	**(0.51–0.93)**
Widowed/divorced/separated	1.53	(0.68–3.41)	**0.56**	**(0.33–0.96)**	1.37	(0.55–3.42)	**0.35**	**(0.17–0.70)**
Household wealth index	**1.77**	**(1.45–2.17)**	0.88	(0.75–1.05)	**1.68**	**(1.32–2.15)**	**0.8**	**(0.66–0.96)**
HIV prevalence	**1.08**	**(1.04–1.12)**	**0.96**	**(0.94–0.97)**	**1.13**	**(1.08–1.18)**	**0.95**	**(0.93–0.97)**
Exposure to mass media	1.42	(0.90–2.23)	0.91	(0.69–1.21)	1.24	(0.77–2.00)	0.92	(0.67–1.26)
Community assignment	**0.06**	**(0.04–0.10)**	**1.78**	**(1.41–2.24)**	0.03	(0.01–0.05)	1.97	(1.50–2.59)
*N*	2226		2944		2226		2944	
	Use treatment services				Use care and support services			
CBOs per 100,000	**1.64**	**(1.51–1.79)**	1.01	(0.94–1.08)	**1.65**	**(1.51–1.80)**	**1.66**	**(1.49–1.19)**
Gender	**2.43**	**(1.48–4.00)**	0.97	(0.67–1.42)	**0.45**	**(0.24–0.84)**	**0.67**	**(0.45–0.99)**
Age	**0.95**	**(0.92–0.98)**	0.98	(0.96–1.00)	1.01	(0.98–1.04)	0.99	(0.97–1.02)
Education								
Secondary	0.99	(0.54–1.82)	0.94	(0.61–1.47)	**3.12**	**(1.40–6.93)**	0.99	(0.54–1.78)
College/university	**2.8**	**(1.40–5.60)**	1.26	(0.77–2.04)	**7.45**	**(3.06–18.14)**	1.71	(0.94–3.11)
Employed	**2.54**	**(1.31–4.91)**	1.02	(0.69–1.51)	**7.29**	**(1.72–30.90)**	1.39	(0.86–2.24)
Marital status								
Married	0.78	(0.44–1.40)	1.13	(0.73–1.73)	1.08	(0.54–2.18)	0.87	(0.54–1.41)
Widowed/divorced/separated	**3.05**	**(1.17–7.94)**	1.58	(0.73–3.34)	1.06	(0.19–5.86)	0.15	(0.02–1.14)
Household wealth index	**1.86**	**(1.31–2.64)**	0.84	(0.64–1.10)	**1.58**	**(1.02–2.46)**	**1.68**	**(1.12–2.05)**
HIV prevalence	1	(0.97–1.04)	0.96	(0.94–0.98)	1.01	(0.96–1.05)	0.99	(0.97–1.02)
Exposure to mass media	**3.65**	**(1.44–9.28)**	1.13	(0.71–1.82)	1.73	(0.63–4.72)	1.22	(0.64–2.35)
Community assignment	**0.49**	**(0.33–0.74)**	1.48	(1.00–2.19)	**0.4**	**(0.27–0.61)**	**0.41**	**(0.25–0.69)**
*N*	2226		2944		2226		2944	

Notes: All coefficients significant at the 95% level are in bold font. The estimated conditional models were specified as follows: DV_ij_ = γ_00_+ß_11_ Age_ij_ + ß_12_ Gender_ij_ + ß_13_Marital status_ij_ + ß_14_ Empolyment status_ij_+ ß_15_ Exposure to media + ß_16_ Househod wealth index_j_ + γ_01_ CBO density_j_ + γ_02_ Pair-wise assignment_j_ + γ_03_HIV Prevalence_j_ + u_0j_ + r_ij_ where DV_ij_ is the dependent variable from ith individual in the *j*th community; γ_00_ is the non-random intercept term; ß_ij_ is the coffecient for the ith individual-level variable from *j*th community; γ_0j_ is the coefficient for the *j*th community-level variable; u_0j_ is the community-level residual; and r_ij_ is the individual-level residual.

## Discussion

The findings suggest that the strength of CBO engagement has a potential to increase the availability and utilization of HIV/AIDS-related services. The CBOs in the evaluation communities focused and spent the highest percentage of their funds on prevention activities as well as on providing support for PLWHA and OVC. Consequently, respondents in the communities with stronger CBO engagement were more likely to say that prevention and care services were available in their community and to report using any HIV/AIDS related services, prevention-related services, and care related services than their counterparts in the communities where CBO engagement was weaker. These findings support the assumption of the Nigerian government and its international partners that CBOs add value to the national response to HIV/AIDS and suggest that CBO engagement strengthens that response by increasing access to and utilization of services. This seems to be especially true in rural areas, where few public or private health care facilities are located. In the absence of other service providers, CBO engagement is particularly important in improving service availability and utilization in rural areas.

Previous studies conducted in Nigeria assessed the impact of specific interventions implemented at the community level but not necessarily by CBOs (see e.g., Arnold et al., 2012; Okanlawon & Asuzu, 2011; Omorodion et al., 2012). To our knowledge this is a first study that examined in a systematic way the impact of CBOs on the availability and utilization of HIV/AIDS-related services at the community level. An evaluation conducted in Kenya using a methodology similar to ours found no association between the strength of CBO engagement measured as the percentage of the population within a community who were aware of CBOs’ work, and reported availability and utilization of services (Riehman et al., 2013). One reason behind the different results may be the different way in which CBO engagement was conceptualized and operationalized (perception of CBO activity versus number of CBOs per 100,000 population). It is also possible that CBOs in some countries are more effective than in others due to social, economic, organizational, or other contextual factors. Future studies should examine such crosscountry differences.

This evaluation should be considered a first step in the continuous assessment of the impact of community engagement on the response to HIV/AIDS in Nigeria. As such, it underscores the need to collect information about CBO involvement in the response to HIV/AIDS in a systematic way on an ongoing basis. In-depth interviews with CBO staff showed that community-based-organizations did not have well established communication channels with national, state, and local bodies coordinating the response to HIV/AIDS in Nigeria. Furthermore, none of the interviewed CBOs conducted a community needs assessment to inform what activities and interventions they would carry out. This is not surprising, given the overall scarcity of financial and human resources those organizations had at their disposal. The activities in which the CBOs engaged were selected based on the founders’ perception of what was needed in the community, which might or might not have reflected true community needs and the local epidemic dynamics. For example, it is striking that while commercial sex workers (CSWs) were identified as most-at-risk population in 7 communities, only one CBO listed CSWs as its target population. Also, the fact that 30% of the total CBO expenditure was allocated to program management and capacity building suggests that CBO resources might not have been allocated efficiently. Future research should address the extent to which community engagement in the response to HIV/AIDS matches local needs and to assess what support strategies (e.g., capacity building, technical assistance) can help ensure that the activities in which CBOs engage are efficient and effective in the local contexts in which the CBOs operate.

One important limitation of the evaluation is that it is based on a cross-sectional design. Therefore, it cannot establish whether the associations we found between CBO engagement and availability and utilization of services is causal, that is, whether CBO efforts actually increased services availability and utilization in their communities. It is possible, that CBOs tended to locate and work in communities where availability and utilization of services were already high. Interviews with CBO staff, however, show that the interviewed CBOs were formed or started working in their communities because of the needs perceived by the founding members, not because of high availability or utilization of health and HIV/ADIS-related services. Nevertheless, in order to further ascertain the causal impact of CBOs on service availability and utilization a systematic over time assessment is necessary. Another limitation is that our measures of service availability and utilization are based on self-reports and may be subject to recall or social desirability bias. Finally, the results described earlier are directly representative only of the 28 communities where the evaluation was conducted. Nevertheless, the communities and the states included in the evaluation represent all geopolitical zones of Nigeria which helped this evaluation consider, albeit in the cursory manner, the cultural, social, and economic diversity of that country.

### Conclusions and implications

Better coordination and closer working relationships between the HIV/AIDS coordinating agencies and CBOs working on the ground can help them leverage and share their resources and expertise and provide a better understanding of CBO needs in terms of funding, capacity building, and training and technical assistance. Establishing of mechanisms to monitor the activities of CBOs (e.g., national or state-level CBO databases), may also encourage the documentation and dissemination of best practices as well as uptake of evidence-based interventions.
